# Renoprotective Effect of Taxifolin in Paracetamol-Induced Nephrotoxicity: Emerging Evidence from an Animal Model

**DOI:** 10.3390/jcm12030876

**Published:** 2023-01-22

**Authors:** Ismail Topal, Mustafa Yaşar Özdamar, Tulin Catakli, İsmail Malkoc, Ahmet Hacimuftuoglu, Charalampos Mamoulakis, Aristidis Tsatsakis, Konstantinos Tsarouhas, Christina Tsitsimpikou, Ali Taghizadehghalehjoughi

**Affiliations:** 1 Department of Pediatric Diseases, Medical Faculty, Erzincan University, 24000 Erzincan, Turkey; 2 Department of Pediatric Surgery, Faculty of Medicine, Erzincan University, 24000 Erzincan, Turkey; 3 Department of Pediatrics, Lokman Hekim Hospital, 06930 Ankara, Turkey; 4 Department of Anatomy, Faculty of Medicine, Ataturk University, 25240 Erzurum, Turkey; 5 Department of Medical Pharmacology, Medical Faculty, Duzce University, 81620 Duzce, Turkey; 6 Department of Urology, University General Hospital of Heraklion, Medical School, University of Crete, 715 00 Heraklion, Greece; 7 Department of Forensic Sciences and Toxicology, Faculty of Medicine, University of Crete, 71003 Heraklion, Greece; 8 Department of Cardiology, University Hospital of Larissa, Terma Mezourlo, 413 34 Larissa, Greece; 9 General Chemical State Laboratory of Greece, 11521 Athens, Greece; 10 Department of Pharmacology, Faculty of Medicine, Seyh Edebali University, 11230 Biecik, Turkey

**Keywords:** acute kidney injury, kidney, nephropathy, paracetamol, renal injury, taxifolin, toxicity

## Abstract

Background: Taxifolin (TXF) is a flavonoid found abundantly in citrus/onion. Encouraging results on its renoprotective effect have been reported in a limited number of drug-induced nephrotoxicity animal models. The present study aimed to evaluate for the first time the potential renoprotective effects of TXF in a paracetamol (PAR)-induced nephrotoxicity rat model. Methods: Rats were divided into three equal groups (*n* = 6 animals per group). Group 1 (PAR group, PARG) received PAR diluted in normal saline by gavage (1000 mg/kg). Group 2 (TXF group, TXFG) received TXF diluted in normal saline by gavage (50 mg/kg) one hour after PAR administration. Group 3 (control group, CG) received normal saline. Twenty-four hours after PAR administration, all animals were sacrificed using high-dose anesthesia. Blood samples were collected and kidneys were removed. Results: The serum blood urea nitrogen, creatinine levels and serum malondialdehyde levels were significantly increased in the PARG. The serum glutathione peroxidase, glutathione reductase and total glutathione levels were significantly higher in the TXFG. At the same time, the kidneys of the PARG animals demonstrated tubular epithelium swelling, distension and severe vacuolar degeneration. The kidneys of the TXFG animals showed mildly dilated/congested blood vessels. Conclusions: The TXF renoprotective effects are promising in preventing PAR-induced nephrotoxicity, mainly through antioxidant activity, and warrant further testing in future studies.

## 1. Introduction

Paracetamol (4′-hydroxyacetanilide, N-acetyl-p-aminophenol, acetaminophen, PAR) is an analgesic-antipyretic drug sold worldwide without prescription in most countries [1]. It is an effective and useful drug at therapeutic doses; however, severe side effects have been reported at high doses [2]. Both isolated renal failure and renal failure combined with liver failure have been reported in association with PAR overdosing [3]. Existing guidelines still encourage treatment based mostly on the dose calculated in the 1970s for the reported studies of acetylcysteine in PAR toxicity [4]. A massive PAR overdose causing nephrotoxicity could occur after extrapolated 4 h plasma PAR concentration (about >250 mg/L) or the intravenous administration of a 150 mg/kg bolus dose [5]. While PAR has been administered by gavage at 0.5–2.5 g/kg to create hepatotoxicity and nephrotoxicity in rats, exact known toxic doses remain inconclusive in the international literature [6].

Acute tubular necrosis and acute renal failure due to PAR toxicity may develop [7]. The elevation of serum creatinine (sCr) and blood urea nitrogen (BUN) levels may be indicators of PAR-induced acute tubular necrosis. Approximately 1–2% of patients exposed to PAR overdose develop renal failure [8,9]. Paracetamol induces liver injury due to its metabolic conversion to the highly reactive intermediate N-acetyl p-benzoquinonimine (NAPQI) by cytochrome P-450 mediated oxidases [10]. This mechanism has been accepted for PAR-induced nephrotoxicity too [11]. Renal toxicity is mediated through NAPQI formation after PAR conjugation through glucuronidation and sulphation is saturated [12]. A PAR overdose causes systemic toxicity by disrupting the balance between NAPQI and basal glutathione levels [2,13]. Accumulated toxic metabolites cause subendothelial damage and acute tubular necrosis. Apart from direct nephrotoxicity, oxidative stress and an insufficient amount of renal glutathione contribute to renal injury and progressing renal failure [14,15,16].

Nowadays, natural antioxidants, rather than chemical drugs, are used as protective agents against organ damage in many diseases [17]. Polyphenols are herbal agents abundant in marine pine bark and contain some antioxidant flavonoids [18,19]. Despite the fact that data regarding the biological functions of polyphenols are abundant, evidence is still inadequate to support the clear beneficial effects on human chronic diseases [20]. Nevertheless, from these antioxidant flavonoids, for example, the protective effect of proanthocyanin and silymarin against renal damage has previously been reported [21,22]. Flavonoids exhibit antioxidant activity by inhibiting the lipid peroxidation and enzymatic reactions responsible for the formation of free radicals. Taxifolin (3, 5, 7, 3, 4-pentahydroxy flavanone or dihydroquercetin, TXF) is a flavonoid found abundantly in citrus and onion [23]. Recently, the protective effects of TXF on hepatotoxicity-induced liver injury have been demonstrated [24]. Encouraging results on the renoprotective effect of TXF have been reported in a limited number of drug-induced nephrotoxicity animal models [25,26,27]. The present study aimed to evaluate for the first time the potential renoprotective effects of TXF in a PAR-induced nephrotoxicity rat model.

## 2. Materials and Methods

### 2.1. Animals

Eighteen adults male Wistar rats (aged 8–10 weeks) weighing 255 ± 15 g were obtained from Atatürk University Medical Experimental Practice and Research Center (Erzurum, Turkey). They were bred and housed in ventilated rooms at a temperature of 24 ± 2 °C, with a 12 h light/dark cycle and a humidity of 60 ± 4%. Thiopental sodium (Pental Sodium, IE Ulagay Drug Co., Istanbul, Turkey), TXF (Evalar, Hertz, Moscow, Russia) and PAR (Parol, Atabay Drug Co., Istanbul, Turkey) were purchased. Rats were randomly divided into three equal groups (*n* = 6 animals in each group). Group 1 (PAR group, PARG) received PAR diluted in normal saline by gavage (1000 mg/kg suspension) at a single dose. Group 2 (TXF group, TXFG) received TXF diluted in normal saline by gavage (50 mg/kg suspension) one hour after PAR administration. Group 3 (Control group, CG) received normal saline only. Twenty-four hours after PAR administration, all animals were sacrificed using high-dose anesthesia (50 mg/kg thiopental), blood samples were collected and kidneys were removed. Prerequisites for the experimental process were in accordance with the Guide for the Care and Use of Laboratory Animals of Atatürk University. The Ethical Committee approved the study (Protocol; 2018/8812460-00.99-EE.45082).

### 2.2. Blood Analyses and Histopathological Evaluation

Serum creatinine (sCr) and blood urea nitrogen (BUN) levels were determined using COBAS Integra 800 analyzer (Roche, Schaffhausen, Switzerland). Oxidative stress biomarkers were measured in the animals’ renal tissues as previously described using renal tissue homogenate (diluted 1:2) [28]. Total plasma protein was determined using a Bradford reagent (Sigm, Rockville, USA). More specifically, malondialdehyde (MDA) was measured in 532 nm wavelength and the pink complex formed at a high temperature (95 °C) based on spectrophotometric measurement [29]. The total glutathione (tGlu) assay (yellow color) was measured at 412 nm wavelength [30]. Glutathione peroxidase (GPO) and Glutathione reductase (GR) activity were determined at 340 nm wavelength (Lawrence and Burk for GPO and Carlberg and Mannervik for GR were used) [31,32]. Rat kidneys were removed immediately after sacrifice. They were fixed in neutral formalin 10% (Sigma, Rockville, USA) solution for 24 h, then embedded in paraffin wax and sectioned (4 µm thickness) for histopathological assessment. Renal tissue sections were stained with hematoxylin and eosin (H and E stain) using a standard protocol and were evaluated under light microscopy [33,34,35].

### 2.3. Statistical Analysis

Statistical analysis was performed using the SPSS software (SPSS Inc., Version 25.0. Chicago, IL, USA). Groups were compared using one-way analysis of variance (ANOVA), following normality/homoscedasticity checks with Shapiro–Wilk test/Leven’s test, respectively. Post hoc comparisons were performed using Tukey/Tamhane’s test, as appropriate. Bivariate correlation analysis was used to find correlations among the biochemical markers monitored both in serum and tissue. The chi-square (*χ*^2^) test was used for the analysis of associations between histopathological findings and levels of oxidative stress markers in tissue samples and sCr. *p* value ≤ 0.05 was considered significant.

## 3. Results

The results of the biochemical analysis in the PARG, TXFG and CG animals, in serum and tissue samples, are summarized in Table 1. Serum creatinine and BUN levels increased following PAR administration compared to the CG; this increase was statistically significant in the PARG and reached over 600% for sCr and over 300% for BUN levels, while in the TXFG the increase in sCr was 11% (nearly significant, *p* = 0.076) and for BUN was insignificant and less than 10%. Oxidative stress markers in renal tissue samples were severely altered after PAR administration. Malondialdehyde, one of the final products of polyunsaturated fatty acid peroxidation in the cells, was measured at more than 2 times higher in the PARG animals compared to the CG. Taxifolin administration almost restored MDA levels compared to the CG (16% increase). Similarly, GSH levels diminished by 63% in the PARG compared to the CG, which points to elevated cellular vulnerability towards oxidative stress and was restored by 83% in the TXFG. The same pattern was observed for GPO and GRx. A non-consistent and weak pattern of bivariate correlations between the biochemical markers monitored was observed within the experimental groups of the present study. In the PARG animals, the renal tissue levels of MDA were positively correlated with GPO (r = 0.890, *p* = 0.046), while GPO kidney levels were negatively correlated with GRx levels in the TXFG animals (r = −0.868, *p* = 0.025). Serum creatine and BUN did not correlate with any tissue oxidative stress markers monitored in all groups, with the exception of sCr; its mildly elevated levels in the TXFG animals were negatively correlated with GPO (r = 0.920, *p* = 0.013).

**Figure 1:**
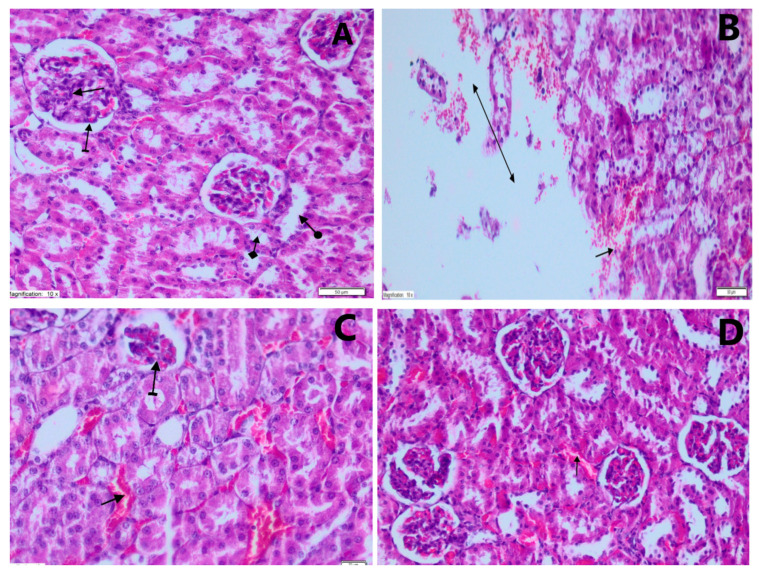
Results of the histopathological analysis. (**A**) Histological status in the control group (CG) animals, without any signs of kidney damage (H&EX200): glomerular structure (straight arrow), bowman capsule (straight arrow), proximal tubule (check arrow) and distal tubule (round arrow). (**B**) Histopathological changes of damaged kidney tissue in the paracetamol group (PARG) (H&EX200): hemorrhage in the parenchyma (straight arrow), destruction and edema (double-sided arrow). (**C**) Histopathological appearance of damaged kidney tissue in the PARG (H&EX200): mild atrophic glomerulus (striated arrow), dilated and congested blood vessels (straight arrow). (**D**) Histopathological changes (H&EX200) in the kidneys of animals treated with Taxifolin after administration of high dose PAR (TXFG): mildly dilated and congested blood vessels (straight arrow).

Kidney specimens demonstrated tubular epithelium swelling, distension and severe vacuolar degeneration in the PARG. Kidney specimens showed mildly dilated and congested blood vessels in the TXFG. No abnormal morphological changes were observed under light microscopy in the kidney specimens of the CG, which received normal saline only. The histopathological results are presented in Figure 1 and their rating is summarized in Table 2.

Serum creatine increase was associated with the severity of glomerular damage (PARG: *p* = 0.015; TXFG: *p* = 0.022). On the other hand, only in the PARG was the MDA increase associated both with the extent of parenchyma destruction and edema (*p* = 0.01) and glomerular damage (*p* = 0.02). Hyperemia in the PARG animals was nearly significantly associated with the decreased GSH levels (*p* = 0.057).

## 4. Discussion

The present study aimed to evaluate for the first time any potential renoprotective effects of TXF in a PAR-induced nephrotoxicity rat model. Potential protective agents previously studied in experimental nephrotoxicity models using PAR high-dosing schemes follow different modes of action and the results remain controversial [36,37,38]. Acetaminophen-related kidney dysfunction has been defined in clinical practice based on the laboratory examination of sCr, BUN, the glomerular filtration rate and albumin sCr ratio [39,40]. In our study, after PAR administration, sCr and BUN levels severely increased; TXF administration counteracted this increase to a large extent for both markers of renal function. Previous studies reported a protective effect of rhein, silymarin and grape seed pro-anthocyanidin on renal function in PAR toxicity and ischemia-reperfusion injury [26]. There are also reports that high sCr, BUN and MDA levels due to nephropathy were significantly decreased after flavonoid administration. In this relation, Baponva et al. (2022), showed that the use of the aqueous extract of the African plant *Amblygonocarpus andongensis*’s stem bark helped recovery from hepatic and renal failure caused by PAR toxicity [9,41].

After orally receiving PAR, nearly 63% of PAR is metabolized through glucuronidation and 34% via sulphation mainly in the liver [42,43]. N-acetyl p-benzoquinonimine plays a role as a reactive intermediate, when the oxidization of 55% of PAR occurs by the microsomal P-450 enzyme system. Taking into consideration PAR metabolism and excretion, changes and the aggravation of the oxidative stress status are expected, not only regarding circulating oxidative stress, but also on a tissue level. N-acetyl p-benzoquinonimine is detoxified by intracellular GSH, when receiving PAR in therapeutic doses. Therefore, serum tGSH levels during nephrotoxicity following PAR overdosing have been recognized as a biochemical marker indicating the degree of kidney injury in animal models. There are also reports of PAR renal toxicity without liver toxicity [42,43]. In our study, although the mean tGSH in the TXFG still remained slightly lower than the CG, the alleviating effect of TXF is evident, similar to other reports on flavonoid administration (Nigella Sativa, Rhein) in order to treat PAR toxicity [44,45].

In the case of kidney damage caused by chemicals and drugs, the inability to eliminate free radicals after oxidative stress results in cellular destruction [46]. Therefore, the local changes in the oxidative status observed could also entail kidney histopathological alterations. In the current study, histopathology showed the presence of hemorrhage, destruction and edema of the renal parenchyma as previously described [42]. Moreover, a mild atrophic glomerulus and dilated/congested blood vessels in kidney specimens of the PARG were found. The subendothelial damage shown—acute tubular necrosis caused by PAR overdose—was documented through elevated sCr and BUN levels. Taxifolin administration restored to a large extent even the histopathological alterations due to PAR toxicity.

Injury to the nephrocellular membrane causes lipid peroxidation, leading to the release of inflammatory mediators or free radicals. Malondialdehyde has been widely accepted as an indicator of the degree of lipid peroxidation [47,48,49]. In our study, mean renal tissue MDA levels were significantly higher in the PARG compared to the CG and the TXFG. Tissue MDA levels after TXF administration returned to CG levels. Our work was consistent with previous studies [50].

Free radicals oxidize biomolecules including proteins, lipids and DNA. The enzyme GPO is a selenium-dependent enzyme and its main effect is the removal of H_2_O_2_, and also it inhibits the formation of highly reactive hydroxyl radicals [29,51]. Glutathione reductase is also a crucial enzyme and serves to restore cellular glutathione levels by reducing oxidized disulfide-glutathione [31]. The mechanism of nephrotoxicity is closely related to the depletion of the antioxidant defense system [52,53,54]. In the present study, GRx and GPO activities were monitored and our findings further support the results of previous experiments demonstrating the protective antioxidant activity of flavonoids, such as TFX, in the PAR nephrotoxicity experimental model [55,56,57].

The limitation of the current study is the use of a single and not escalating dosing scheme for TFX after PAR nephrotoxicity is established. Furthermore, different time intervals for administering TXF could be studied, such as pre-conditioning with TXF [39] and simultaneous administration with PAR, in order to mimic hospitalization treatment conditions. Finally, a comparative study of other antioxidants with TFX in the said model could further demonstrate the nephroprotective effectiveness of TXF.

## 5. Conclusions

Taxifolin has been accepted as an essential component of some dietary supplements and antioxidant-rich functional foods. Moreover, the antioxidant activity of TXF has been previously demonstrated by the ferric thiocyanate method [24,27,28,31,43]. The present study, for the first time, evaluated the potential renoprotective effects of TXF in a PAR-induced nephrotoxicity rat model. Oxidative damage was proven as a component of PAR nephrotoxicity. Our results provide evidence that TXF may have the potential to prevent PAR-induced nephrotoxicity through its antioxidant activity and warrants further testing in future studies and clinical trials.

## Figures and Tables

**Table 1:** Levels of Malondialdehyde (MDA), total Glutathione (tGSH), Glutathione peroxidase (GPO) and Glutathione reductase (GRx) monitored in renal tissues samples, serum Creatinine (sCr) and blood urea nitrogen (BUN) from the Paracetamol group (PARG), the Taxifolin group (TXFG) and the Control (CG).

Parameter Monitored	PARG	TXFG	CG
**MDA (μmol/gr protein)**	6.86 ± 0.212 ^##^	3.58 ± 0.242 **	3.08 ± 0.303
**tGSH (nmol/gr protein)**	2.84 ± 0.324 ^##^	6.88 ± 0.422 **	7.70 ± 0.683
**GPO (U/gr protein)**	3.00 ± 0.421 ^##^	6.95 ± 0.693 **	8.18 ± 0.491
**GRx (U/gr protein)**	3.51 ± 0.441 ^##^	8.21 ± 0.402 **	8.96 ± 0.512
**sCr (mg/dL)**	2.73 ± 0.282 ^##^	0.414 ± 0.0532 **	0.373 ± 0.0532
**Blood Urea Nitrogen (mg/dL)**	158 ± 7.55 ^##^	37.3 ± 2.80 **	34.0 ± 2.75

^##^: comparison between PARG and CG, *p* < 0.001. **: comparison between PARG TXFG, *p* < 0.001. No statistical difference was detected between TXFG and CG.

**Table 2:** Summary of the histopathological scores based on H and E staining results for kidney tissues.

Histopathological Findings	Control Group	PARG	TXFG
**Glomerular Damage**	-	+++ (6/6 animals)	+ (4/6 animals)
**Parenchyma destruction and edema**	-	+++ (6/6 animals)	+ (4/6 animals)
**Hyperemia**	-	+++ (6/6 animals)	++ (5/6 animals)

Control: healthy group (no treatment), PARG: Paracetamol group, TXFG: Taxifolin group. Histopathological findings rating grades: None (-), mild (+), moderate (++), severe (+++).

## Data Availability

Raw data is available on request.
